# A systematic review to identify assessment instruments for social isolation or loneliness in adults with heart failure

**DOI:** 10.1038/s44325-025-00044-y

**Published:** 2025-03-07

**Authors:** Scott William, Julee McDonagh, Sabine M Allida, Ayele Semachew Kasa, Christopher Patterson, Hiba Deek, Lorna Moxham, Timothy Wand, Caleb Ferguson

**Affiliations:** 1 Centre for Chronic and Complex Care Research, Blacktown Hospital, Western Sydney Local Health District, Blacktown, NSW Australia; 2 School of Nursing, Faculty of Science, Health and Medicine, University of Wollongong, Wollongong, NSW Australia; 3 Nursing Department, Faculty of Health Sciences, Beirut Arab University, Beirut, Lebanon

**Keywords:** Cardiology, Health care

## Abstract

Social isolation and loneliness are frequently associated with heart failure. It is unclear how these constructs are assessed in adults living with heart failure which warrants further exploration. This review aimed to identify how social isolation and loneliness is assessed in adults living with heart failure. This is a systematic review reported according to PRISMA and registered in Prospero on 18 March 2024 [CRD42024518571]. The bibliographic databases, MEDLINE, CINAHL, and Scopus were searched from inception to 20 March 2024. Original quantitative studies assessing loneliness and/or social isolation of adults living with heart failure using a patient-reported instrument and written in English language were included. The Joanna Briggs Institute Critical Appraisal checklists were used to assess the quality of included studies. The results were presented narratively. Thirty studies (17 cohort studies, 9 cross-sectional studies, 2 RCTs, and 2 case control) with 529,665 participants (mean age ranged from 52 to 83 years, 57% were women) were included. The University of California Los Angeles Loneliness Scale was the most commonly used instrument to assess loneliness while composite measures of network size and frequency of social contacts were the most commonly used to assess social isolation in adults living with heart failure. Social isolation and loneliness exert deleterious effects on both mental and physical health, significantly diminishing life satisfaction. The improved use of social isolation and loneliness assessment instruments may contribute to more effective interventions, ultimately enabling care that may enhance the health outcomes and quality of life of adults living with heart failure.

## Background

Heart failure is an enduring and debilitating syndrome, affecting 65 million people world-wide^1^. The prognosis for heart failure is worse than many cancers^2^. People living with heart failure experience symptoms such as breathlessness, limited exercise capacity, fatigue, edema, disrupted sleep patterns, which can contribute to poor quality of life^3^. The management of heart failure is complex, often punctuated by frequent hospitalization and emergency department visits. Further, coping and adjusting to living with heart failure can be challenging for the individual affected, but also family caregivers who provide care at home, or in the community. Multimorbidity, frailty, depression, anxiety, social isolation, and loneliness are commonly associated with heart failure, contributing to worse patient outcomes and increased costs^4–6^. High quality social connections and community interactions are important to optimize the well-being of adults living with heart failure^7^.

Loneliness is a subjective experience and occurs when the social connections and individual needs in life are greater than the connections they have^8^. Social isolation does not discriminate, it is estimated to impact 1 in 4 older adults^9^. Recognized as a modern-day health crisis, it is associated with increased mortality and as a risk factor comparable to smoking, obesity, and physical activity^9^. Such concern is well founded given the links that social isolation and loneliness has to mental illness, emotional distress, suicide, the development of dementia, premature death, and poor health behaviors including smoking, physical inactivity, and poor sleep and can influence physiological outcomes including high blood pressure and impaired immune function^10^. The COVID-19 pandemic, generational shifts, a lack of social and community cohesion and housing shifts to apartment living are identified as a few contributing factors to the worsening impacts^11–14^.

Social isolation is defined as the objective state of having a small network of kin and non-kin relationships and this few or infrequent social interactions^15^. Whereas loneliness is defined as a painful subjective feeling that results from a discrepancy between desired and actual social connections^9^. Social isolation has been reported as a strong determinant of health and predictor of mortality^16^. Social isolation and loneliness have increasingly been suggested as risk factors for cardiovascular disease^17^. A UK-based cohort study in 2023 involving 12,898 participants with heart failure, examined the relationship with loneliness and social isolation^18^. The study identified a significant association between the factors, with the impact of social isolation on heart failure potentially modified by loneliness status (P-interaction = 0.034)^18^. Interventions that focus on individual and communal experiences of social isolation and loneliness could indeed be measures for the maintenance of cardiovascular health^18^. The risk associated with social isolation are not restricted to certain areas, cultures or sociodemographic as this was shown in a recent systematic review outlining the high rates of loneliness across high, middle, and low-income countries^19^. Whilst often used interchangeably, social isolation and loneliness are conceptually different. Importantly, socially isolated people may not necessarily be lonely and vice versa. Therefore, assessing loneliness and social isolation is crucial in this context.

Social isolation and loneliness are social determinants that clinicians need to recognize and address as important components of patient centred health care^17^. Therefore, should be regularly evaluated for all patients particularly those with cardiometabolic disease who are at high risk of developing heart failure^17^. To date, it is unclear how social isolation and loneliness have been assessed in adults living with heart failure, this warrants further exploration.

The primary aim of this review was to identify patient reported assessment instruments used to access social isolation and loneliness in adults living with heart failure.

## Methods

### Design

A systematic review was conducted and reported in accordance with the Preferred Reporting Items for Systematic Reviews and Meta-Analyses (PRISMA) statement, see Supplementary Materials 1 and 2^20^. The systematic review protocol was registered prospectively in PROSPERO on the 18^th^, March 2024 RECORD ID: [CRD42024518571].

The participants, phenomena of interest, and the context (PICo) approach was used to guide formulation of the search strategy and research question for this systematic review^21^. Participants: adults living with heart failure, phenomena of interest: patient reported social isolation and/or loneliness, context: original research studies including a predominant population of adults living with heart failure using a patient reported assessment instruments to measure social isolation and/or loneliness.

This review included primary observational cohort studies, randomized controlled trials (RCT), quasi-experimental single group pre-/post- studies assessing loneliness and/or social isolation using a patient reported assessment instrument. Conference abstracts, reviews, editorials, and any publications not written in English or secondary analyses were excluded.

### Eligibility criteria

Original studies that included adults with a primary or secondary diagnosis of heart failure, consistent with heart failure guidelines were included^22–25^. Studies that did not investigate heart failure or did not include a predominant population living with heart failure (≥20%) were excluded.

This review included proportions of adults living with heart failure assessed for loneliness and/or social isolation using a patient reported assessment instrument. Studies that did not use a patient reported assessment instrument, i.e., electronic health record derived status as lived alone were excluded.

A search of three electronic bibliographic databases MEDLINE, CINAHL, and Scopus was conducted. Databases were searched from inception to 20^th^ March 2024. Only papers written in English language were included. The search strategy was reviewed by experts in the fields of heart failure and mental health. The detailed search strategy is provided in Table 1 in the appendix.

**Table 1:** Search strategy

Database	Search terms
Medline	(heart failure) or (CCF) or (Cardiomyopathy) or (Congestive HF) or (cardiac failure) AND ((Loneliness) OR (Social Isolation))
Scopus	TITLE-ABS-KEY((“heart failure” OR “Cardiomyopathy” OR “Congestive heart failure” OR “Cardiac Failure”) AND (“Loneliness” OR “Social Isolation”))
CINAHL	((heart failure) OR (CCF) OR (Cardiomyopathy) OR (Congestive heart failure) OR (Cardiac Failure)) AND ((Social Isolation) OR (Loneliness))

The search results were downloaded and uploaded to Covidence^26^ for screening. Two reviewers independently conducted the title and abstract and full text, and data extraction. Conflicts were resolved by a third independent reviewer.

Potential overlaps between studies were identified at full text review to prevent double counting individual patients. This was done by comparing the study country, location, setting (hospital/community), and participant sample size. For instance, if two studies were from the same country, location, and used the same assessment instruments for social isolation and/or loneliness, only the study most recently published was included.

The following data were extracted using a standardized data extraction form on Covidence:

- Publication details: first author, journal, year, title, lead author contact details, country where study was conducted.
- Characteristics of included studies: methods, aim of study, study design, funding source, possible conflicts of interest.
- Participants: population description, inclusion criteria, exclusion criteria, prevalence of social isolation and loneliness, patient data, recruitment method.
- Patient reported assessment instruments: for social isolation and loneliness, type(s) of assessment instrument(s), if the study used a qualitative, quantitative, or mixed method assessment.
- Outcome data: mean scores or number of people and percentage of people with social isolation and/or loneliness, if reported.

### Quality appraisal

Quality assessment was performed by two reviewers independently. The Joanna Briggs Institute (JBI) critical appraisal assessment instruments were adopted to assess the methodological quality and risk of bias using applicable scoring for the study types: RCT (0–13), cross sectional studies (0–8), cohort studies (0–11), and case control studies (0 to 10)^27–29^. A score was assigned for each item from zero for “No” or “Unclear” responses and a score of one for a “Yes” response. The scores of the items for each study were summed to obtain a total quality score. Quality of the studies was then classified into three categories according to the JBI instrument used, these categories were low-quality (high risk of bias) when the quality appraisal score ranged from 0 to 4, moderate quality (moderate risk of bias) from 5 to 7, and high quality (low risk of bias) from eight and above. Studies having low and moderate risk of bias were included^30–32^. All studies scored within the respective ranges for low and moderate risk of bias according to study type and therefore none were excluded at quality appraisal. Disagreements between reviewers were resolved through discussions or consultation with a third independent reviewer.

### Analysis

There were no quantitative analyses performed. The results are presented as a narrative summary description of the individual studies and outcomes guided using the principles of the Cochrane framework for narrative data synthesis and analysis^33^.

## Results

**Fig. 1:**
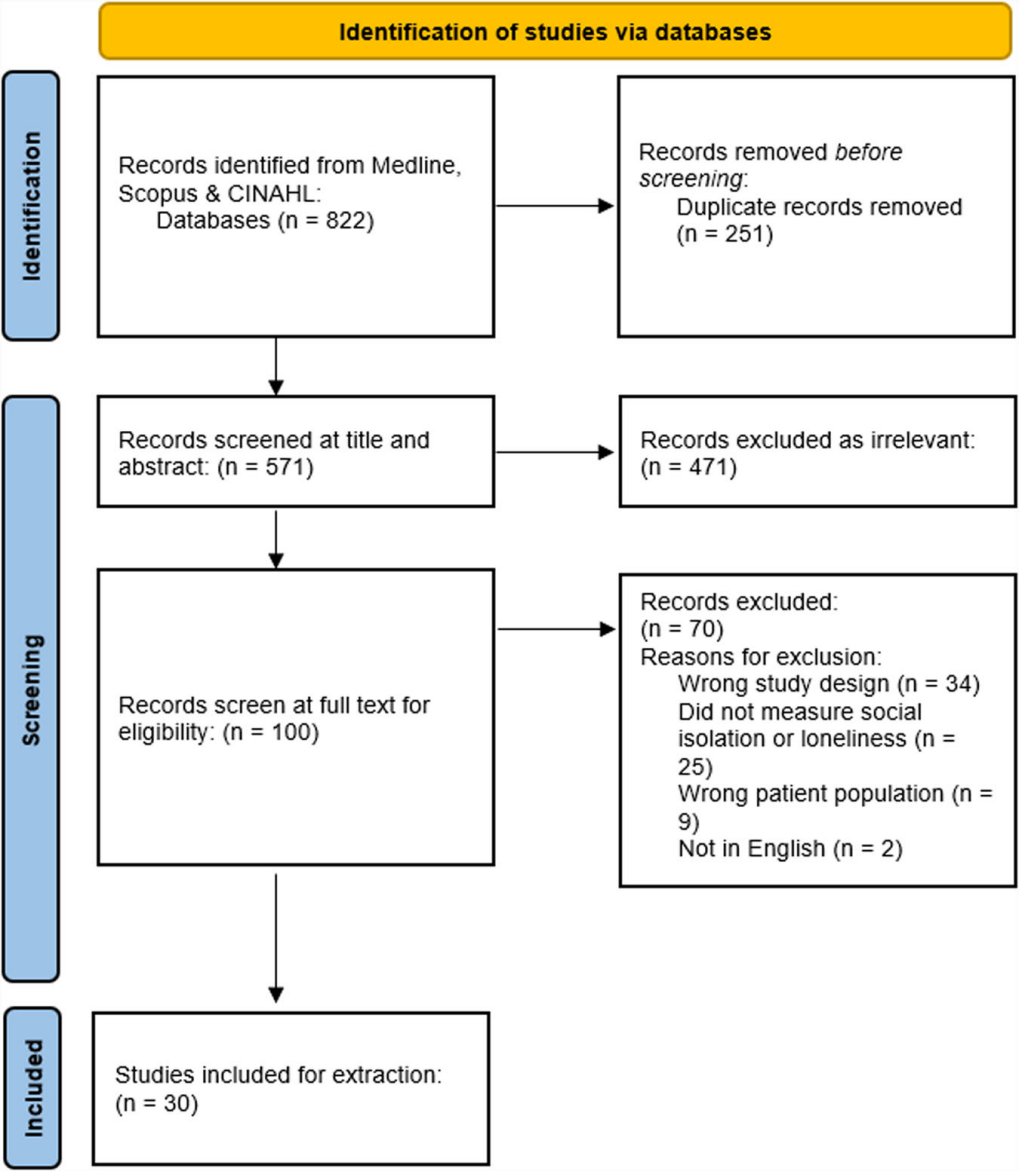
The Preferred Reporting Items for Systematic Reviews Meta-Analyses flow diagram.

A total of 822 articles were retrieved from the three databases, of which 251 duplicates were removed. We screened 571 titles and abstracts and excluded 471 as irrelevant. Full text screening was conducted for 100 articles, of which 70 were excluded with reasons. Refer to Fig. 1 for the PRISMA diagram and the reasons for exclusion. In total, 30 studies were included for data extraction.

There was a total of 17 cohort studies, nine cross-sectional studies, two RCTs, and two case control studies included in this review. The total number of participants across the included studies was *n* = 529,665 mean age ranged from 52 to 83 years, 57% (*n* = 303,046) were women.

The studies originated from 15 countries (14 studies included participants from the United States, two from Sweden, two from Japan, two from the United Kingdom, one from Germany and Austria. The other remaining countries were single sites. Refer to Table 2 for the study characteristics and Table 3 for the outcomes.

**Table 2:** Summary table of included studies

Author/Year/Country	Study design	Aim	Participants/setting
(Riegel and Carlson^56^) United States	Randomized controlled trial	“To test the acceptability and effectiveness of a peer support intervention for patients hospitalized with HF.”	Patients in clinic
(Deka et al.^53^) United States	Randomized controlled trial	“To compare the experimental and comparison groups on adherence to recommended exercise guidelines (150 min/week of moderate intensity exercise); intention to adhere to recommended exercise guidelines; and outcomes including: functional status, self-efficacy for exercise adherence, and perceived social isolation.”	Patients in clinic
(Nichols et al.^54^) United States	Cross sectional study	“To compare self-reported domains of HF-related health between patients with a HF diagnosis who had and had not experienced a recent HF hospitalization.”	Patients in clinic
(Liang et al.^18^) United Kingdom	Cohort study	“To examine the association of social isolation, loneliness, and their combination with incident HF.”	Community dwelling
(Savitz et al.^48^) United States	Cohort study	“To address the gaps of the co-occurrence of multiple Social Risk Factors (SRFs) and the association with health care utilization using latent class analysis (LCA) as a novel approach to understand the clustering of SRFs.”	Community dwelling
(Yildirim et al.^60^) Turkey	Cohort study	“To investigate the correlation between death anxiety, loneliness and hope levels in patients receiving treatment in a cardiac intensive care unit (ICU).”	Patients in clinic
(Brouwers et al.^61^) The Netherlands	Cohort study	“To examine the relative importance of these models as underlying etiological factors of depression in HF patients by using a cross-sectional and prospective measurement approach.”	Patients in clinic
(Obiegło et al.^52^) Poland	Cross sectional study	“To analyse an association between these two variables in a large group of individuals with at least a 6-month history of heart failure.”	Patients in clinic
(Athilingam et al.^62^) United States	Cross sectional study	“To examine the association between elevated cytokines, C-reactive protein (CRP), and cognition in HF.”	Patients in clinic
(Longman et al.^50^) Australia	Cross sectional study	“This paper aims to describe characteristics of older, rural patients frequently admitted with ACS conditions and identify factors associated with their admissions from the patient perspective.”	Patients in clinic
(Keyes et al.^49^) United States	Cohort study	“To identify the relationship between social isolation levels and earlier (i.e., less than 30-day post-discharge) and later (i.e., 30 days or later) hospital readmission rates in a convenience sample of geriatric CHF patients.”	Patients in clinic
(Murberg^34^) Norway	Cohort study	“To evaluate the possible effects of social relationships (perceived social support and perceived social isolation) on mortality risk in 119 patients with stable, symptomatic congestive heart failure.”	Patients in clinic
(Griffin et al.^63^) United States	Cross sectional study	“To identify demographic factors associated with loneliness and to examine the relationship between loneliness and both stress and depression after LVAD.”	Patients in clinic
(Löfvenmark et al.^57^) Sweden	Cohort study	“To investigate perceived loneliness and social support in patients diagnosed with CHF. To investigate whether loneliness and perceived social support may be associated with gender, age, healthcare utilization, and mortality.”	Patients in clinic
(Manemann et al.^47^) United States	Cohort study	“To determine, among a community cohort of patients with HF, whether perceived social isolation, as measured by a brief screener that could be easily incorporated into clinical practice to identify at-risk patients, is associated with death and health-care use, including hospitalizations, emergency department (ED) visits, and outpatient visits.”	Community dwelling
(Polikandrioti^59^) Greece	Cross sectional study	“To evaluate the factors associated with perceived social isolation and to assess the impact of fatigue on social isolation.”	Patients in clinic
(Allemann et al.^44^) Sweden	Cross sectional study	“To explore factors related to perceived social support in a large cohort of individuals with HF living with an ICD or a cardiac resynchronization therapy defibrillator (CRT-D).”	Patients in clinic
(Seo et al.^45^) United States	Cross sectional study	“To test a structural equation model (SEM) of the predictors of cognitive/affective and somatic depression, including dyspnea, family support, friend support, and loneliness, after controlling for age and gender.”	Patients in clinic
(Rocha et al.^64^) Uruguay	Case control study	“To examine predictors of drop out from a multidisciplinary heart failure program.”	Patients in clinic
(Yang et al.^39^) China	Cross sectional study	“To examine the multiple mediation effects of activities of daily living and social isolation on the relationship between physical symptoms and loneliness in patients with heart failure.”	Patients in clinic
(Spaderna et al.^35^) Germany and Austria	Cohort study	“To evaluate depression and social isolation assessed at time of waitlisting as predictors of survival in heart transplant (HTx) recipients.”	Patients in clinic
(Sterling et al.^36^) United States	Cohort study	“To determine factors independently associated with 30-day all-cause readmission among Medicare beneficiaries hospitalized for HF in the REGARDS study (Reasons for Geographic and Racial Differences in Stroke).”	Community dwelling
(Zhang et al.^38^) United States	Cohort study	“To determine whether social determinants of health (SDOH) are associated with cardiologist involvement in the management of adults hospitalized for HF.”	Community dwelling
(Kitakata et al.^40^) Japan	Cohort study	“To elucidate the prevalence and prognostic impact of the risk for SI and patient preferences regarding ACP and EOL care among hospitalized patients with HF at risk for SI.”	Patients in clinic
(Cené et al.^42^) United States	Cohort study	“To examines whether social isolation is associated with incident HF in older women and examines depressive symptoms as a potential mediator and age and race and ethnicity as effect modifiers.”	Community dwelling
(Saito et al.^41^) Japan	Cohort study	“To investigate the prevalence of social isolation and its association with rehospitalization in patients with HF in the Asian population.”	Patients in clinic
(Cené et al.^43^) United States	Cohort study	“To prospectively examine whether individuals at higher vs. low risk for social isolation have higher HF incidence in the Atherosclerosis Risk in Communities (ARIC) study; To determine whether the association between social isolation and incident HF is mediated by vital exhaustion.”	Patients in clinic
(Coyte et al.^37^) United Kingdom	Cohort study	“To investigate the association between social relationships, captured through multiple measures, and heart failure incidence, whilst adjusting for biological and behavioral covariables in a community-dwelling sample of older British men.”	Community dwelling
(Checa et al.^51^) Spain	Cohort study	“To determine the impact of social context on mortality in patients with advanced HF.”	Patients in clinic
(Dickson et al.^46^) United States	Case Control Study	“To describe the cultural beliefs about self-care. To identify social factors that facilitate or impede HF self-care, and to explore how these sociocultural factors influence self-care practices among blacks with HF.”	Patients in clinic

**Table 3:** Summary table of outcomes of included studies

Author/Year	Participant characteristics	Social Isolation/Loneliness Results	Measure of Social Isolation and/or loneliness	Did the study use a validated measure?	Domain of measure
(Riegel and Carlson^56^)	Mean age: Usual care: 73.28 years (SD 13.09) Intervention: 72.64 (SD 13.0) Total: 72.95 (SD 12.97) Number of participants: Usual care: 43 Intervention group: 45 Total: 88 Females: 51 (58%) NYHA classification: I—4.5% II—31.8% III—44.4% IV—19.3% Comorbidity Scores: Low 42% Med 36.4% High 21.6%	“Minor difference reported in the intervention group (*F* = 5.94, *P* = 0.004). No significant group differences in heart failure readmissions, length of stay, or cost were evident at 90-days were reported.”	SI: Seventeen items from the UCLA social Support Inventory	Yes	SI: Which support is sought and received and satisfaction with the support: Information or advice, tangible assistance or aid, and emotional support (Dunkel-Schetter et al.^95^).
(Deka et al.^53^)	Mean age: Experimental: 61.7 (SD 11.3) Comparison: 67.7 (SD 11.4) Total: 64.7 (SD 11.5) Number of participants: Experimental: 15 Comparison: 15 Total: 30 Females: 11 NYHA classification proportions: I—3, II—17, III—10	“Overall Video session attendance was 68%, with 73% of participants attending five or more sessions. Adherence to exercise was 58.8% in the experimental group and 57.3% in the comparison group. The experimental group perceived receiving social support through the internet-based synchronized face-to-face video meetings but due to a small sample size and lack of adequate power, no significant impact on exercise adherence was observed. Participants commented that feedback regarding physical activity from the Fitbit Charge HR was helpful and motivational.”	SI: Friendship scale for perceived social isolation.	Yes	SI: Social connection, relatability, loneliness and contact with others for emotional support. (Hawthorne^96^)
(Nichols et al.^54^)	Mean age: 74.8 (SD 10.3) *N* = 468 Females: 243 (52%) LVEF < 40% = 3.7% 41–49% = 2.3% >50% = 50.7%	“After two mailings, we received 468 completed questionnaires for response rate of 23.4%. Patients with a recent HF hospitalization had significantly lower scores on the KCCQ-12 Quality of Life (52.6 vs. 59.6, *p* = 0.016) and Social Limitations (48.4 vs. 55.5, *p* = 0.009) scales as well as the Clinical Summary Scale (50.8 vs. 55.3, *p* = 0.048) and Total KCCQ-12 score (49.6 vs. 56.8, *p* = 0.003). In sequential logistic regression models designed to achieve parsimony, Total KCCQ was a strong predictor of being in the recent hospitalization group. When using the KCCQ-12 sub-scales, the Social Limitations scale was a strong predictor of being in the recent hospitalization group.”	SI: Kansas City Cardiomyopathy Questionnaire Social Limitations Domain	Yes	This instrument is used for heart failure patients exclusively and measures the impairment heart failure causes the patient to partake in several social activities (Green et al. ^55^).
(Liang et al.^18^)	Mean age: 56.5 (SD 8.1) Females: 254230 (54.7%) *N* = 464,773 Lived alone: 85,991	“Among the 464,773 participants (mean age: 56.5SD 8.1 years, 45.3% male), 12,898 incident HF cases were documented during a median follow-up of 12.3 years. Social isolation (most vs least: adjusted HR: 1.17; 95% CI:1.11–1.23) and loneliness (yes vs no: adjusted HR: 1.19; 95% CI: 1.11–1.27) were significantly associated with an increased risk of incident HF. The association between an elevated risk of HF and social isolation was modified by loneliness (P-interaction ¼ 0.034). A gradient of association between social isolation and the risk of incident HF was found only among individuals without loneliness (P-trend < 0.001), but not among those with loneliness (P-trend ¼ 0.829). These associations were independent of the genetic risk of HF.”	SI: Modified version of the Berkman-Syme Social Network Index. LO: Loneliness was assessed with 2 questions that were derived from the UCLA-LS	No	SI: Social network and contact’s index (Berkman and Syme^75^). LO: Loneliness (Russell^69^).
(Savitz et al.^48^)	Mean age: 73.4 (SD 12.0) Females: 1419 (45.2%)	“Latent class analysis was used to identify subgroups of SRFs; associations with outcomes were examined. A total of 3142 patients with HF (mean age, 73.4 years; 45% women) had SRF data available. The SRFs with the strongest association with hospitalizations were education, social isolation, and area‐deprivation index. We identified 4 groups using latent class analysis, with group 3, characterized by more SRFs, at increased risk of emergency department visits (hazard ratio [HR], 1.33 [95% CI, 1.23–1.45]) and hospitalizations (HR, 1.42 [95% CI, 1.28–1.58])”	LO: PRO-MIS Social Isolation Short Form 4a v2.0	Yes	LO: Loneliness (Cella et al.,^72^; Organization^73^; PROMIS Health Organization,^74^.
(Yildirim et al.^60^)	Mean age: 63.56 (SD 12.74) *N* = 150 Females: 59 (39.3%)	“The patients had a mean age of 63.56 ± 12.74 years. Most of the patients (82%) were treated in the ICU for heart failure. There was a statistically significant. positive correlation between total scores of TDAS and UCLA-LS (r = 0.337; *p* < 0.001) and a statistically significant negative correlation between total scores of UCLA-LS and HHI (*r* = 0.292; *p* < 0.001). Also, there was a statistically significant negative correlation between the scores of UCLA-LS and Positive Readiness and Expectancy Subscale (*r* = 0.164; *p* = 0.044). The multiple linear regression indicated that the model was statistically significant (*F* = 7.177, *p* < 0.001). The variables of age and UCLA-LS among those included in the model were statistically significant predictors of the death anxiety scores of the patients (23.1%) (*p* < 0.05).”	LO: UCLA-LS adapted into Turkish language	Yes	LO: Emotional loneliness, social loneliness, and existential loneliness (Russell^69^).
(Brouwers et al.^61^)	Mean age: 66.7 (SD 8.7) *N* = 268 Females: 66 (24%) NYHA Classification: I/II—237 (90.5%) III—31 (10%)	“At baseline, NYHA class, body mass index, educational level, Type D personality and loneliness were significantly associated with depression. Higher NYHA class (*B* = 2.25; SE = 0.83), higher educational level (*B* = 1.41; SE = 0.48), Type D personality (*B* = 2.56; SE = 0.60) and loneliness (B = .19; SE = .05) were also independently associated with higher depression levels at one-year follow-up (all *p* < .005). Inflammation, brain natriuretic peptide and left ventricular ejection fraction were not related to depression over time.”	LO: UCLA-LS	Yes	LO: emotional loneliness, social loneliness, and existential loneliness (Russell^69^).
(Obiegło et al.^52^)	Mean age: 62.3 (SD 12.2) *N* = 100 Females: 32 (32%) NYHA Classification: II—10, III—54, IV—36	“The patients presenting with low levels of acceptance of illness (8–18 points) scored significantly higher on the energy, pain, emotional reaction, sleep, social isolation and mobility domains of the NHP. Multivariate analysis showed that acceptance of illness was the only independent predictor of quality of life in all the NHP domains: energy (*β* = −0.653, *p* < 0.001), pain (*β* = −1.464, *p* < 0.001), emotional reactions (*β* –1.738, *p* < 0.001), sleep (*β* = −0.820, *p* < 0.001), social isolation (*β* = −0.638, *p* < 0.001) and mobility (*β* = −1.739, *p* < 0.001). Male gender proved to be an independent predictor of lower pain scores (*β* = −1.320, *p* = 0.001) and divorce was associated with higher social isolation scores (*β* = 1.948, *p* < 0.001).”	SI: Nottingham Health Profile (NHP) questionnaire sub-scale for social isolation	Yes	SI: Social isolation (Wiklund^97^).
(Athilingam et al.^62^)	Mean age: 61.7 (SD 8.8) *N* = 38 Females: 12 (31.6%) LVEF Mean 27.7% NYHA Classification: I—4 (10.5%), II—20 (52.6%), III—14 (36.7%) Lived alone: 6 (15.8%)	“In the multiple linear regressions, the covariate set consisting of education, living arrangements, and UCLA loneliness score explained 46% of the total variation in MoCA scores (Table IV). When IL-6 was added to the model, an additional 11% of the variation in MoCA score was explained and IL-6 was independently associated with MoCA score (*P* < 0.001). Parameter estimates of the covariates and IL-6 (log-transformed) are provided (Table V). IL-6 was strongly but inversely associated with MoCA score after controlling for covariates education, living arrangements, and UCLA loneliness score. When IL-6, TNF-a, and CRP were entered stepwise, the model was significant”	LO: UCLA-LS	Yes	LO: Emotional loneliness, social loneliness, and existential loneliness (Russell^69^).
(Longman et al.^50^)	Mean age: 77.1 (range 66–95) *N* = 102 Females: 39 (16%) Comorbidity scores: 0–28 (27.5%) 1-2–55 (53.9%) >3–19 (18.6%) Lived alone: 36 (35%)	“Survey respondents (*n* = 102) had a mean age of 77.1 years (range 66–95 years), and a mean of 4.1 admissions within 12 months; 49% had at least three chronic conditions; the majority had low socioeconomic status; one in five (22%) reported some difficulty affording their medication; and 35% lived alone. The majority reported psychological distress with 31% having moderate or severe psychological distress. While all had a GP, only 38% reported having a written GP care plan. 22% of those who needed regular help with daily tasks did not have a close friend or relative who regularly cared for them. Factors independently associated with more frequent (*n* = 4+) relative to less frequent (*n* = 3) admissions included having congestive heart failure (*p* = 0.003), higher social isolation scores (*p* = 0.040) and higher Charlson Comorbidity Index scores (*p* = 0.049). Most respondents (61%) felt there was nothing that could have avoided their most recent admission, although some potential avoidability of admission was described around medication and health behaviors. Respondents were younger and less sick than non-respondents.”	The Duke Social Support Index using four items on the size of respondent’s social networks and amount of social con-tact.	Yes	Social networks and loneliness index (Koenig et al.,^80^; Landerman et al.^79^).
(Keyes et al.^49^)	Mean age: Early Readmissions: 83.3 Late and Non-Readmissions: 82.4 *N* = 286. Females Early Readmissions: 52 (55.3%) Late and Non-Readmissions: 121 (63.0%) Total sample: 173 (60.45%) Lived alone: 53	“There were no statistically significant differences between earlier hospital readmissions versus later/non-readmitted sample patients when grouped by age, race, gender, or level of measured social isolation. However, composite comorbidity scores were significantly lower for patients in the >30-day/non-readmitted subgroup compared to earlier readmission patients.”	SI: Self-reported status of: Currently unmarried,2. Lives alone, and 3. Lacks caregiver. LO: Measured as a “Yes” or “No” responses	No	SI: Perceived support network. LO: Loneliness.
(Murberg^34^)	Mean age: 66 (SD 9.1) *N* = 119 Females: 34 (28.6%) NYHA Classification: I—2 (1.7%), II—71 (59.7%), III—43 (36.1%), IV—3 (2.5%)	“Fifty-one deaths were registered during the six-year follow-up period, all from cardiac causes. Analysis using proportional hazard models indicated that social isolation was a significant predictor of mortality (relative risk, 1.36; confidence interval, 1.04–1.78; *p* < 0.03), controlling for neuroticism, heart failure severity, functional status, gender, and age. The small sample size was a limitation of the study; therefore, further research is required in order to confirm these findings and to illuminate the mechanisms behind the relationships between social isolation and mortality.”	SI: Social isolation was assessed on the basis of four items.Perceived social support was assessed by a 15-item scale.	No	Perceived social support, and Social Isolation with contact with family, other relatives, and friends.
(Griffin et al.^63^)	Mean age: 61.75 (SD 12.76) *N* = 73 Females = 15 (20.55%)	“Loneliness was measured via the loneliness item from the Center for Epidemiologic Studies Depression (CESD), depression via the CESD (excluding the loneliness item), and stress via the Perceived Stress Scale. In bivariate analyses, older age (OR per year = 0.958, 95%CI = 0.919–0.998) and being partnered (OR = 0.245, 95%CI = 0.083–0.724) were associated with less loneliness. In the multivariate model, there was an interaction effect between age and partnership (*p* = 0.0212), where older age was protective against loneliness for non-partnered, but not partnered, patients. Higher loneliness was associated with higher stress (*β* = 0.484, *B* = 5.687, 95%CI = 3.195–8.178) and depression (*β* = 0.618, *B* = 7.544, 95%CI = 5.241–9.848). Patients who are not partnered and younger may be at increased risk of loneliness after LVAD.”	LO: Loneliness was assessed using an item from the Center for Epidemiologic Studies Depression Scale (CES-D)	Yes	LO: Loneliness (Radloff^98^; Shaffer^99^).
(Löfvenmark et al.^57^)	Mean age: 76 (SD 10.3) *N* = 146 Female: 71 NYHA Classification: I—18(15%), II—42 (36%), III 25 (21%), IV 1 (1%) Lived alone: 62	“Loneliness was reported by 29 (20%) participants. They were more often women (*p* = 0.001) and younger (*p* = 0.024). Patients who perceived loneliness had fewer social contacts (*p* = 0.033), reported lower occurrence of emotional contacts (*p* = 0.004), were less satisfied with social contacts and close relationships (*p* = 0.001). Those reporting loneliness had more days hospitalized (*p* = 0.044), and more readmissions to hospital (*p* = 0.027), despite not having more severe CHF.”	SI: The Interview Schedule for Social Interaction (ISSI) was used to measure perceived social support. LO: Measured by one single-item question.	Yes	ISSI: To assess the availability and perceived adequacy for different domains of social relationships. Availability of social integration, availability of attachment, adequacy of social integration, and adequacy of attachment (Henderson et al.^100^). LO: Loneliness.
(Manemann et al.^47^)	Mean age: 73.3 *N* = 1681 Females: 783 (46.60%)	“A total of 2003 patients returned the survey (response rate, 52%); 1681 patients completed all questions and were retained for analysis. Among these patients (53% men; mean age, 73 years), 19% (*n* = 312) had moderate perceived social isolation and 6% (*n* = 108) had high perceived social isolation. After adjustment, patients reporting moderate perceived social isolation did not have an increased risk of death, hospitalizations, or emergency department visits compared with patients reporting low perceived social isolation; however, patients reporting high perceived social isolation had >3.5 times increased risk of death (hazard ratio, 3.74; 95% confidence interval [CI], 1.82–7.70), 68% increased risk of hospitalization (hazard ratio, 1.68; 95% CI, 1.18–2.39), and 57% increased risk of emergency department visits (hazard ratio, 1.57; 95% CI, 1.09–2.27). Compared with patients who self-reported low perceived social isolation, patients reporting moderate perceived social isolation had a 16% increased risk of outpatient visits (rate ratio, 1.16; 95% CI, 1.03–1.31), whereas those reporting high perceived social isolation had a 26% increased risk (rate ratio, 1.26; 95% CI, 1.04–1.53).”	LO: PRO-MIS Social Isolation Short Form 4a v2.0	Yes	LO: Loneliness (Cella et al.,^72^; Organization^73^; PROMIS Health Organization,^74^.
(Polikandrioti^59^)	Mean age: 68.6 (SD 7.1) *N* = 100 Females: 32 (32%) NYHA Classification: II—20 (20%), III—36 (36%), IV 44 (44%)	“Of the 100 participants (68% men; mean age, 68.6 ± 7.1 years), 78% reported perceiving social isolation. Factors significantly associated with perceived social isolation were female sex (*P* = 0.001), New York Heart Association class IV (*P* = 0.001), stress about HF (*P* = 0.002), paroxysmal nocturnal dyspnea (*P* = 0.030), edema in the lower limbs (*P* = 0.001), report of receiving many medications (*P* = 0.001), change in body image (*P* = 0.032), and not following limitations in fluid and sodium intake (*P* = 0.001). The MFIS total score determined moderate to high levels of fatigue (median, 70 points; range, 21–105 points). Total fatigue was statistically significantly associated with social isolation as perceived by patients (*P* = 0.001).”	SI: Self-report item of whether they perceived social isolation on a 4-point Likert scale (1 = very much, 2 = enough, 3 = a little, and 4 = not at all).	No	SI Perception of social isolation.
(Allemann et al.^44^)	Mean age: 67.3 (SD 9.8) *N* = 1550 Females: 303 (19.5%) Lived alone: 331 (21.5%)	“Social Isolation Score 5.96 (SD 1.2) Most reported a high level of social support, but 18% did not. In logistic regression, living alone was the greatest predictor of low/medium support. Lower social support for those living alone was associated with poorer perceived health status, having symptoms of depression, and experiencing low perceived control. For those living with someone, lower support was associated with female gender, symptoms of depression and anxiety, and less control. Heart failure status and perceived symptom severity was not related to the outcome.”	SI: MSPSS	Yes	SI: Perception of support from the domains of friends, family, and significant others (Zimet et al.^65^).
(Seo et al.^45^)	Mean age: 57.19 (SD 13.38) *N* = 151 Female: 74 (49.3%) New York Heart Association Classification: II—89 (59.3%), III/IV—55 (36.7%), IV—6 (4.0%) Lived alone: 38 (25%)	“Structural equation modeling (SEM) showed that cognitive/affective depression was predicted by greater dyspnea and loneliness, whereas somatic depression was predicted by more dyspnea and friend support. Also, greater dyspnea was related to more loneliness and less friend support; less friend support was related to loneliness. Women reported more dyspnea and loneliness.”	SI: MSPSS LO: Loneliness was measured by one item from the Duke Social Support Index	Yes	SI: Perception of support from the domains of friends, family and significant others (Zimet et al.^65^). Duke: Network size and contact’s index (Koenig et al.^80^; Landerman et al.^79^).
(Rocha et al.^64^)	Mean age: Cases: 67 (SD 14) Controls: 70 (SD 9) Females:Cases: 4 (%) Controls: 12 (%) Total sample: 16 (%) NYHA Classification:Cases: I—3 (21%), II—7 (50%), III—4 (29%) Control: I—18 (43%), II—19 (45%), III—5 (12%) T: I—21 (%), II—26 (%), III 9 (%) Lived alone: 13 Living alone was 35.7% in dropouts.	“The only significant factor associated with dropout was social isolation. Patients who lived alone, without family support, had a significantly greater dropout risk (odds ratio, 12.5; 95% confidence interval, 1.35–11.6)”	LO: Lives alone SI: Support from family members	No	LO: Loneliness SI: Family support
(Yang et al.^39^)	Mean age: 69.67 (SD 8.02) Females: 143 (47.2%) *N* = 303 NYHA Classification: II—156 (51.5%), III/IV—147 (48.5%) Lived alone: 20 (6.6%) Number of comorbidities <3129 (42.6%) ≥3174 (57.4%)	“Of the 303 patients, 66.7% experienced mild loneliness and 21.8% experienced moderate or severe loneliness. Multiple mediation analysis showed that physical symptoms had a direct effect on loneliness (effect ¼ 0.210; 95% confidence interval (CI) 0.0990.320) and the link between physical symptoms and loneliness through 3 indirect pathways^1^: activities of daily living (effect ¼ 0.043; 95% CI 0.006‒0.086), accounting for 20.48% of the total effect^2^; social isolation (effect ¼ 0.060; 95% CI 0.005‒0.120), accounting for 28.57% of the total effect; and^3^ activities of daily living and social isolation in series (effect ¼ 0.049; 95% CI 0.024‒0.081), accounting for 23.33% of the total effect. The total mediating effect was 72.38%”	SI: LSNS-6. LO: UCLA-LS	Yes	SI: Measures perceived social support received by friends and family as a social network index (J. Lubben et al.^78^,). LO: emotional loneliness, social loneliness, and existential loneliness (Russell^69^,).
(Spaderna et al.^35^)	Mean age: 52.2 *N* = 148 Females: 27 (18.20%) Comorbidity Scores: Previous heart surgery (*n* = 126) 40 (27.0%) Atrial fibrillation (*n* = 109) 21 (14.2%) ICD (*n* = 118) 81 (54.7%) Lived alone 28 (18.9%)	“Higher depression scores increased the risk of dying (hazard ratio=1.07, 95% confidence interval, 1.01, 1.15, *P* = 0.032), which was moderated by social isolation scores (significant interaction term; hazard ratio = 0.985, 95% confidence interval, 0.973, 0.998; *P* = 0.022). These findings were maintained in multivariate models controlling for covariates (*P* values 0.020–0.039). Actuarial 1-year/5-year survival was best for patients with low depression who were not socially isolated at waitlisting (86% after 1 year, 79% after 5 years). Survival of those who were either depressed, or socially isolated or both, was lower, especially 5 years posttransplant (56%, 60%, and 62%, respectively).”	SI: Composite measure of social isolation defined as frequency of contact with friends or family	No	Network size and contact’s index.
(Sterling et al.^36^)	Mean age: 76^71–82^ *median (IQR) Females: 306 (44.3%) LVEF < 50 = 280 (55%) Comorbidity Scores: 4.0^3–5^ *median (IQR)	“We assessed 9 SDOH based on the Healthy People 2030 Framework: race, education, income, social isolation, social network, residential poverty, Health Professional Shortage Area, rural residence, and state public health infrastructure. The primary outcome was 30-day all-cause readmission. For each SDOH, we calculated incidence per 1000 person-years and multivariable-adjusted hazard ratios of readmission. Among 690 participants, the median age was 76 years at hospitalization (interquartile range, 71–82), 44.3% were women, 35.5% were Black, 23.5% had low educational attainment, 63.0% had low income, 21.0% had zip code–level poverty, 43.5% resided in Health Professional Shortage Areas, 39.3% lived in states with poor public health infrastructure, 13.1% were socially isolated, 13.3% had poor social networks, and 10.2% lived in rural areas. The 30-day readmission rate was 22.4%. In an unadjusted analysis, only Health Professional Shortage Area was significantly associated with 30-day readmission; in a fully adjusted analysis, none of the 9 SDOH were individually associated with 30-day readmission.”	SI: Composite measure of social isolation defined as frequency of contact with friends or family	No	Network size and contact’s index.
(Zhang et al.^38^)	Mean age: 77.8 (71.5-84) *median (IQR) *N* = 1000 Females: 479 (47.9%) NYHA Classification: I—57 (7.1%), II—237 (31.1%), III—303 (39.7%), IV—169 (22.1%)	“Of the 303 patients, 66.7% experienced mild loneliness and 21.8% experienced moderate or severe loneliness. Multiple mediation analysis showed that physical symptoms had a direct effect on loneliness (effect ¼ 0.210; 95% confidence interval (CI) 0.0990.320) and the link between physical symptoms and loneliness through 3 indirect pathways^1^: activities of daily living (effect ¼ 0.043; 95% CI 0.006‒0.086), accounting for 20.48% of the total effect^2^; social isolation (effect ¼ 0.060; 95% CI 0.005‒0.120), accounting for 28.57% of the total effect; and^3^ activities of daily living and social isolation in series (effect ¼ 0.049; 95% CI 0.024‒0.081), accounting for 23.33% of the total effect. The total mediating effect was 72.38%.”	SI: Composite measure of social isolation defined as frequency of contact with friends or family.	No	Network size and contact’s index.
(Kitakata et al.^40^)	Mean age: 73.0 Female: 31 (35.8%) *N* = 120 Lived alone: 24	“A Cox proportional hazard model was constructed to elucidate the short-term (180-day) prognostic impact of SI. Overall, 28.3% of participants were at high risk for SI (6-item Lubben Social Network Scale score <12). High‐risk patients had more negative attitudes toward ACP than those without (61.8% versus 80.2%; *P* = 0.035). The actual performance of ACP conversation in patients with and without high risk were 20.6% and 30.2%, respectively. Regarding preference in end‐of‐life care, “Saying what one wants to tell loved ones” (73.5% versus 90.6%; *P* = 0.016) and “Spending enough time with family” (58.8% versus 77.9%; *P* = 0.035) were less important in high‐risk patients. High risk for SI was associated with higher 180‐day risk‐adjusted all‐cause mortality (hazard ratio, 7.89 [95% CI, 1.53–40.75]).”	SI: LSNS-6	Yes	Measures perceived social support received by friends and family as a social network index (J. Lubben et al.^78^,).
(Cené et al.^42^)	Mean age: 62.6 *N* = 36457 Females: 36457 (100%) Lived alone: Reported as Hazard ratios	“Over a median follow‐up of 15.0 years, we analyzed data from 36 457 women, and 2364 (6.5%) incident HF cases occurred; 2510 (6.9%) participants were socially isolated. In multivariable analyses adjusted for sociodemographic, behavioral, clinical, and general health/functioning; socially isolated women had a higher risk of incident HF than nonisolated women (HR, 1.23; 95% CI, 1.08–1.41). Adding depressive symptoms in the model did not change this association (HR, 1.22; 95% CI, 1.07–1.40). Neither race and ethnicity nor age moderated the association between social isolation and incident HF.”	Berkman-Syme Social Network Index.	No	Network size and contacts index with intimate contacts, religion, and community (Berkman and Syme^75^).
(Saito et al.^41^)	Mean age: 80 (SD 8) *N* = 148 Females: 73 (49%) Living alone: 28 (19%)	“Among 148 patients with heart failure (80 ± 8 years old, 51% male), 73 (49%) were socially isolated. The patients with social isolation had similar comorbidities compared with those without social isolation. Heart failure rehospitalization occurred within 90 days for 25 patients and the heart failure rehospitalization rate was significantly higher in the social isolation group (*p* = 0.036). LASSO (least absolute shrinkage and selection operator) regression confirmed that social isolation was one of the strongest predictors of heart failure rehospitalization, showing larger effects than living alone, being unemployed, and other established risk factors.”	SI: LSNS-6	Yes	Measures perceived social support received by friends and family as a social network index (Lubben et al.^78^,).
(Cené et al.^43^)	Mean age: 56.9 (SD 5.7) *N* = 12995 Females: 7147 (55.0%)	“After a median follow-up of 16.9 person-years, 1727 (13.0%) incident HF events occurred. The adjusted hazard of incident HF was greater for those in the higher vs. low social isolation risk group (hazard ratio 1.21, 95% confidence interval 1.08–1.35). Our data suggest that vital exhaustion strongly mediates the association between higher social isolation and incident HF (the percentage change in beta coefficient for higher vs. low social isolation groups after adjusting for vital exhaustion was 36%).”	SI: LSNS-10	Yes	Perception of social engagement and social support with friends, family, and neighbors as a network index (James Earl Lubben,^77^; James E. Lubben^76^,).
(Coyte et al.^37^)	Mean age: 69.8 (SD 5.4) *N* = 3698 Females: 0% Lived alone: 407 (11.3%)	“Among 3698 participants, 330 developed heart failure. Men with low compared to high frequency of contact with family and friends had an increased risk of incident heart failure [hazard ratio (HR) 1.59, 95% confidence interval (CI) 1.15–2.18]; this remained statistically significant after adjustment for social class, behavioral, and biological risk factors. Low compared to high scores for satisfaction with contacts was associated with increased risk of heart failure (adjusted HR = 1.54; 95% CI 1.14–2.07). Lower social relationship scores (combining frequency and satisfaction with contact) were associated with greater risk of incident heart failure (adjusted HR = 1.38, 95% CI 1.02–1.87). Marital status and living alone were not significantly associated with heart failure.”	SI: A Frequency of Contact score.	No	Network size and contact’s index.
(Checa et al.^51^)	Mean age: 82 (SD 9.0) *N* = 1148 Females: 708 (61.70%) Comorbidity scores: *N* = 155 (13%) Charlson Comorbidity Index > 5 NYHA Classification: IV—1148 (100%)	“Data from 1148 New York Heart Association class IV patients were analyzed. Mean (SD) age was 82 (9.0) years, and 61.7% were women. The mean (SD) follow-up was 18.2 (11.9) months. Mortality occurred in 592 patients. Social risk was identified in 63.6% of the patients, and 9.3% acknowledged having social problems. In the adjusted multivariate model, being male (hazard ratio (HR), 1.82; 95% confidence interval [CI], 1.16–2.83), having high dependency on others for basic activities of daily living (HR, 2.16; 95% CI, 1.21–3.85), and presenting with a social problem (HR, 2.46; 95% CI, 1.22–4.97) were related to an increased risk of mortality.”	SI: Gijon’s Social-Familial Evaluation Scale	Yes	SI: Living situation, family situation, economic status, housing, social relationships, and support networks (González et al.^101^).
(Dickson et al.^46^)	Mean age: 59.63 (SD 15.19) *N* = 30 Females: 12 (40%) NYHA Classification: II—10 (33.3%), III—40 (66.6%)	“Self-care was very poor (standardized mean [SD] Self-care of Heart Failure Index [SCHFI] maintenance, 60.05 [18.12]; SCHFI management, 51.19 [18.98]; SCHFI confidence, 62.64 [8.16]). The overarching qualitative theme was that self-care is influenced by cultural beliefs, including the meaning ascribed to HF, and by social norms. The common belief that HF was inevitable (“all my people have bad hearts”) or attributed to “stress” influenced daily self-care. Spirituality was also linked to self-care (“the doctor may order it but I pray on it”). Cultural beliefs supported some self-care behaviors like medication adherence. Difficulty reconciling cultural preferences (favorite foods) with the salt-restricted diet was evident. The significant relationship of social support and self-care (*r* = 0.451, *P* = 0.01) was explicated by the qualitative data. Social norms interfered with willingness to access social support, and “selectivity” in whom individuals confided led to social isolation and confounded self-care practices.”	SI: MSPSS	Yes	Perception of support from the domains of friends, family, and significant others (Zimet et al.^65^,).

^**^LO loneliness, *SI* social isolation.

The most commonly found measures to assess social isolation are listed here. Five studies used non-validated measurements of social network size and/or frequency of contacts within a specified period to quantify and assess either loneliness or social isolation risk^34–38^. While six studies used either complete or modified versions of the Berkman-Syme Social Network Index and the Lubben Social Network Scale, which provide similar scoring systems to composite measures of network size and a contacts index^18,39–43^. Overall, 11 studies assessed social isolation using network size and frequency of contacts as an index.

The Multidimensional Scale of Perceived Social Support (MSPSS) was used by three studies^44–46^. The Berkman-Syme Social Network Index was used by two studies^18,42^, of which one used only three questions adapted from the Berkman-Syme^18^. The 6-item Lubben Social Network Scale (LSNS-6) was used by three studies of which one used the abbreviated version^39–41^10-item version the of the Lubben Social Network Scale (LSNS-10)^43^. The Patient-Reported Outcomes Measurement Information System Social Isolation Short Form 4a V2.0 (PROMIS) was used by two studies^47,48^. One study assessed social isolation through a scoring system determined by asking the patient if they lived alone, were currently unmarried, and if they lacked a caregiver^49^.

The Duke Social Support Index was used by two studies^45,50^ of which one used only one item^45^ while another used four items from this instrument^50^. The Gijon Familial Evaluation was used by one study^51^ and another study used part of the Nottingham Health Profile Questionnaire to assess social isolation^52^. The Friendship Scale for Perceived Social Isolation was used by only one study^53^. One study used the Social Limitations domain of the Kansas City Cardiomyopathy Questionnaire^54^. This assessment instrument was specifically developed for the heart failure population^55^. The UCLA Social Support Inventory (UCLA-SSI) was used by one study^56^. One study used the interview schedule for social interaction which is a quantitative assessment instrument supplemented by qualitative responses^57^. This study used a non-validated 15-item scale mentioned in a prior study to assess social support^58^, alongside a contacts index^34^. One study used a self-report four-point single item non-validated Likert-scale to assess perception of social isolation from 1 to 4^59^.

The most commonly used validated instrument for loneliness was various versions of the University of California Loneliness Scale (UCLA-LS), used by seven studies^18,39,47,48,60–62^. Two of these seven studies used an adapted version from the PROMIS database^47,48^. A single item from The Centre for Epidemiologic Studies Depression Scale (CES-D) was used by one study^63^. One study used a closed ended question of “are you lonely?” with a yes/no answer to assess loneliness^49^ while another study used a multiple-choice question “does it happen that you feel lonely?” with the four possible answers of “Yes always”, “Yes often”, “No seldom” and “No never”^57^. One study looked at family support constructs alongside a patient reported living alone status^64^. A total of thirteen studies reported data on living alone status as either patient reported or collected through electronic medical records as part of baseline assessments^18,35,37,39,40,42,44,45,49,50,57,62,64^.

### Quality of the included studies

The quality appraisal result demonstrated that one of the RCT studies (Table 4), two of the cross-sectional studies (Table 5), and 14 of the cohort studies (Table 6), two case-control studies design (Table 7) had a high quality (low risk of bias). The rest of the studies in each study design demonstrated a moderate quality (moderate risk of bias).

**Table 4:** Critical appraisal result of the studies using RCT study design

Included articles	Criterion no. (items included to appraise RCT studies)															
	1	2	3	4	5	6	7	8	9	10	11	12	13	Total	Raw %	Risk
(Deka et al.^53^,)	√	√	√	*	*	√	*	¥	¥	¥	√	√	√	7	53.8	Moderate
(Riegel and Carlson^56^,)	√	√	√	*	*	√	√	√	¥	√	¥	√	√	9	69.2	Low

√ = yes, X = no, * = unclear, ¥ = not applicable.

Criterion No. 1: Was true randomization used for assignment of participants to treatment groups? No. 2: Was allocation to treatment groups concealed? No. 3: Were treatment groups similar at the baseline? No. 4: Were participants blind to treatment assignment? No. 5: Were those delivering treatment blind to treatment assignment? No. 6: Were outcomes assessors blind to treatment assignment? No. 7: Were treatments groups treated identically other than the intervention of interest? No. 8: Was follow-up complete, and if not, were strategies to address incomplete follow-up utilized? No 9: Were participants analyzed in the groups to which they were randomized? No. 10: Were outcomes measured in the same way for treatment groups? No. 11: Were outcomes measured in a reliable way? No. 12: Was appropriate statistical analysis used? No. 13: Was the trial design appropriate?.

**Table 5:** Critical appraisal result of the studies using cross-sectional study design

Included articles	Criterion no. (items included to appraise cross-sectional studies)										
	1	2	3	4	5	6	7	8	Total	Raw %	Risk
(Allemann et al.^44^)	√	√	√	√	*	*	√	√	6	75	Moderate
(Athilingam et al.^62^)	√	√	√	√	√	√	√	√	8	100	Low
(Griffin et al.^63^)	*	*	√	√	√	*	√	√	5	62.5	Moderate
(Longman et al.^50^)	√	√	√	√	*	*	√	√	6	75	Moderate
(Nichols et al.^54^)	√	√	√	√	X	X	√	*	5	62.5	Moderate
(Obiegło et al.^52^)	√	√	√	√	X	X	√	√	6	75	Moderate
(Polikandrioti^59^)	√	√	*	√	X	X	√	√	5	62.5	Moderate
(Yang et al.^39^)	√	√	√	√	√	√	√	√	8	100	Low
(Seo et al.^45^)	√	√	*	*	√	√	*	√	5	62.5	Moderate

√ = yes, X = no, * = unclear

Criterion no. 1: inclusion criteria, criterion no. 2: description of study subject and setting, criterion no. 3: valid and reliable measurement of exposure, criterion no. 4: objective and standard criteria used, criterion no. 5: identification of confounder, criterion no. 6: strategies to handle confounder, criterion no. 7: outcome measurement, and criterion no. 8: appropriate statistical analysis.

**Table 6:** Critical appraisal result of the studies using cohort study design

Included articles	Criterion no. (items included to appraise cohort studies)													
	1	2	3	4	5	6	7	8	9	10	11	Total	Raw %	Risk
(Brouwers et al.^61^)	*	X	√	*	*	√	√	√	√	√	√	7	63.6	Moderate
(Cené et al.^43^)	√	*	√	√	√	√	√	√	√	√	√	10	90.9	Low
(Checa et al.^51^)	√	√	√	*	*	√	√	√	√	√	√	9	81.8	Low
(Cené et al.^42^)	√	√	√	*	√	√	√	√	√	√	√	10	90.9	Low
(Coyte et al.^37^)	√	√	√	√	√	√	√	√	√	√	√	11	100	Low
(Liang et al.^18^)	√	√	√	√	√	√	√	√	√	√	√	11	100	Low
(Manemann et al.^47^)	√	√	√	X	*	√	√	√	√	√	√	9	81.8	Low
(Murberg^34^)	√	√	√	X	*	*	√	√	√	√	√	8	72.7	Low
(Savitz et al.^48^)	√	√	√	*	*	√	√	√	√	√	√	8	81.8	Low
(Spaderna et al.^35^)	√	√	X	*	*	√	√	√	√	√	√	8	72.7	Low
(Sterling et al.^36^)	√	√	*	*	*	√	√	√	√	√	√	8	72.7	Low
(Yang et al.^39^)	√	√	X	√	√	√	√	√	√	√	√	10	90.9	Low
(Saito et al.^41^)	√	√	√	*	*	√	√	X	√	√	√	8	72.7	Low
(Keyes et al.^49^)	√	√	X	X	X	√	√	√	*	√	√	7	63.6	Moderate
(Löfvenmark et al.^57^)	√	√	√	*	*	√	√	√	√	√	√	9	81.8	Low
(Kitakata et al.^40^)	√	√	√	√	√	√	√	X	*	*	√	8	72.7	Low
(Yildirim et al.^60^)	√	√	√	√	*	√	√	X	*	*	√	7	63.6	Moderate

√ = yes, X = no, * = unclear, ¥ = not applicable

Criterion No. 1: Were the two groups similar and recruited from the same population? No. 2: Were the exposures measured similarly to assign people to both exposed and unexposed groups? No. 3: Was the exposure measured in a valid and reliable way? No. 4: Were confounding factors identified? No. 5: Were strategies to deal with confounding factors stated? No. 6: Were the groups/participants free of the outcome at the start of the study (or at the moment of exposure)? No. 7: Were the outcomes measured in a valid and reliable way? No. 8: Was the follow up time reported and sufficient to be long enough for outcomes to occur? No 9: Was follow up complete, and if not, were the reasons to loss to follow up described and explored? No. 10: Were strategies to address incomplete follow up utilized? No. 11: Was appropriate statistical analysis used?

**Table 7:** Critical appraisal result of the studies using case-control study design

Included articles	Criterion no. (items included to appraise Case-control studies)												
	1	2	3	4	5	6	7	8	9	10	Total	Raw %	Risk
(Rocha et al.^64^)	√	√	√	√	√	√	√	√	√	√	10	100	Low
(Dickson et al.^46^)	√	*	*	√	√	√	√	√	√	√	8	100	Low

√ = yes, X = no, * = unclear

Criterion no. 1: Were the groups comparable other than the presence of disease in cases or the absence of disease in controls? criterion no. 2: cases and controls matched appropriately? criterion no. 3: same criteria used for identification of cases and controls? criterion no. 4: exposure measured in a standard, valid and reliable way?, criterion no. 5: exposure measured in the same way for cases and controls? criterion no. 6: confounding factors identified, criterion no. 7: strategies to deal with confounding factors stated? criterion no. 8: outcomes assessed in a standard, valid and reliable way for cases and controls? Criteria no. 9: the exposure period of interest long enough to be meaningful? And criteria 10: appropriate statistical analysis used?

## Discussion

This systematic review revealed that there are a range of instruments used to assess social isolation or loneliness of people with heart failure in research studies. A description of the most commonly used (defined as used by two or more included studies) assessment instruments from the included studies is provided below.

*Multidimensional Scale of Perceived Social Support (MSPSS)* was originally developed from a survey study using a cohort of undergraduate students by Zimet et al in 1988 to assess the perception of social support across the domains of family, friends, and significant other. The Cronbach alpha coefficient for significant other, family, and friends were 0.91, 0.87 and 0.85, respectively, indicating moderate to good internal consistency. The test retest for these respective domains and for the entire instrument was 0.72, 0.85, 0.75, and 0.88 which means moderate to good reliability^65^. The assessment instrument was further tested in three groups (in pregnant women, adolescents living in Europe with their families, and pediatric physician residents in training) in 1990. The results demonstrated strong internal consistency with Cronbach alpha coefficients ranging from 0.81 to 0.98 across the subscales and between the study groups^66^. In 2018, a psychometric evaluation of the MSPSS in people living with chronic diseases reported good internal consistency evidenced by a Cronbach coefficient alpha of 0.92 for the family domain, 0.96 for friends 0.93, for significant other and 0.91 for the entire assessment instrument^67^.

*The University of California Loneliness Scale (UCLA-LS)* was originally developed in 1978 as a 20-item scale with high internal validity by Russell, Peplau and Ferguson at the University of California Los Angeles. This measure was developed due to a reported lack of simple and reliable assessment instruments to measure loneliness^68^. The most commonly used version is version 3 developed in 1996 with good internal consistency (Cronbach coefficient alpha ranging from 0.89 to 0.94) and moderate reliability (test-retest reliability, *r* = 0.73)^69^. A systematic review of the psychometric properties of UCLA-LS on adults in variation cross-cultural adaptations, including various short and long forms of the assessment instrument also showed moderate to good internal consistency (Cronbach alpha of 0.76–0.93). Although they found that UCLA-LS versions 4, 6, 7, and 10 had the better internal consistencies^70^. Version 3 of this assessment measure was recently validated on 47 chronic obstructive pulmonary disease patients, demonstrating good validity and test-retest reliability^71^. *The Patient-reported Outcomes Measurement Information System (PROMIS) Social Isolation Short Form 4a v2.0* is another abbreviated version of the UCLA, which uses only four items adapted from the UCLA Version 3 as part of the PROMIS database of patient reported outcome measurements^72–74^.

*Berkman-Syme Social Network Index* was originally developed by Berkman and Syme in 1979 to measure social network indexes for comparison with health outcomes in the general adult population^75^. This assessment instrument uses a scoring of frequency of contact with friends, family, neighbors, community and religious connections to quantify social isolation risk^75^. *The Lubben Social Network Scale 6 and 10 (LSNS-6 and LSNS-10)* is an adaptation of the Berkman-Syme Social Network Index developed by James E. Lubben at UCLA to assess social networks in older adults. They reported an original Cronbach alpha of 0.70 in the 1980s. This was originally a 10-item scale and was developed incorporating the domains of family networks, friend networks, and interdependent networks to assess self-reported perceived support from social networks^76,77^. The LSNS-6 abbreviated version was tested and developed by Lubben in three populations of older European communities, across the three sites of Hamburg, Solothurn, and Lonson. The Cronbach alpha coefficient was reported as 0.83 indicating good internal consistency^78^.

The *DUKE Social Support Index* was originally developed as a 35-item scale in the 1980s by researcher at Duke University^79^. This assessment instrument has further been abbreviated to a 23-item and an 11-item version for use in chronically ill aged populations^80^. The 11-item Duke Social Support Index has been validated in older Australians with Cronbach alpha coefficients ranging from 0.6 to 0.8 indicating moderate internal consistency^81,82^.

*Kansas City Myocardiopathy Questionnaire was* developed in 1996 by Spertus, Green, and colleagues to quantify health outcomes for adults living with congestive heart failure. Therefore, this assessment instrument was specifically validated in a cohort of adults living with heart failure. The (23-item) assessment instrument assessed seven domains of Physical limitation 0.90, Symptoms 0.88, Quality of Life 0.78, Social limitation 0.86, Self-efficacy 0.62, KCCQ functional status 0.93, KCCQ Clinical Summary 0.95 demonstrating good internal consistency in use with adults living with heart failure^55^. This assessment instrument has a 2-week recall period and is commonly used by medical device and drug companies. There are 12-item and 23-item versions available for use^83^.

Current widely accepted measures of loneliness include the variations of the UCLA Loneliness Scale. Social isolation is widely assessed using the various composite measures of social network size and frequency of contacts. It is recognized that while there are accepted assessment measurements of social isolation and loneliness, there are no “gold standard measurements”^17^. Researchers and clinicians, might use different assessment instruments and methods, leading to variations in how social isolation and loneliness are measured and reported. This can lead to challenges when developing and considering interventions for heart failure based on these factors. Additionally, loneliness can be perceived differently by different cultures and societies based on their collectivism/individualism scope. A recent study showed the Global Collectivism Index (GCI) across 188 nations globally to show that among the nations of the current study Turkey had the highest GCI accounting for 0.04 and Greece recording the least GCI of −0.062^84^. With the globalization, societies have been shifting from collectivism to individualism leading to significant changes on the loneliness scores. This was reported in a study comparing Japan and London which showed that the rates of loneliness were much higher in Japan than in London while also increasing over a 6-year period^85^. These rates, and changes, reflect a need for continuous assessment of the perceived loneliness rates among the older adult population, including those with heart failure. In addition to the evaluation of these rates on the outcomes of adults living with heart failure.

A recent systematic review on social isolation, loneliness and the impacts on cardiovascular disease outcomes found that diminished social relationships were associated with up to a 16% increased likelihood of experiencing a cardiovascular event^86^, this is similar to previous research which linked social isolation as a risk factor for experiencing a cardiovascular event^87^. There is consistent evidence that social isolation is a strong determinant of health^16^. Currently, there is a growing public health concern labeled the ‘loneliness epidemic’ notably affecting older adults demonstrating a cause-effect relationship on health outcomes^11^, and also in the context of adults living with heart failure^18^.

Having a sense of purpose, that is, feeling that one’s life is goal-oriented and driven, tends to be according to^88^, protective for psychological health. Ma et al.^89^ with their nationally representative longitudinal panel study of older adults in the United States with a working sample of 2649 concur. In fact, the research team found that purpose in life fully mediated the negative impact of loneliness on protective behaviors when measured cross-sectionally^89^. Loneliness has a direct correlation with social connectedness and in this regard and of foci in this paper, there is a well-documented, strong association between social relationships and cardiometabolic disease^17,90^. There could be justification in examining the roles of biomarkers as indicators of social isolation and/or loneliness in patients.

Social isolation and loneliness are different constructs and have a close relationship. However, they are often discussed together though it is important to note that socially isolated people are not necessarily lonely, and lonely people are not necessarily socially isolated in an objective sense^91^. Social isolation refers to the objective concerns and characteristics of a situation and refers to the absence of relationships with other people. It is the state of having few or no social relationships or infrequent social interactions. Loneliness though is a subjective feeling of being alone or disconnected, regardless of the actual amount of social contact. Given the differences the two constructs are measure differently. Social isolation is usually assessed numerically by the number of social contacts a person has, the frequency of social interactions, and the size of their social network. Loneliness, however, is most often assessed using self-report scales where the person rates their feelings of loneliness and perceived social support on validated assessment instruments such as those identified in this paper.

The role of proxy social isolation and loneliness measurements in the clinical setting needs further exploration. This could take the form of composite, validated, quality or single item patient reported assessment instruments. Is asking if somebody is feeling lonely an appropriate use of clinical resources, will the use of proxy measures in clinical practice provide adequate insights to predict patient outcomes?

Do validation measures have justification or is it justifiable to use non-validated measures specific to the context of a patient population? These could be number of interactions, how many visits, social network size, or simple questions such as “*do you feel lonely?*” or “*are you socially isolated*?” or “*do you feel you receive sufficient social support?*”.

Given the impact of social isolation and loneliness on individual and population mental health and wellbeing, any study that improves understanding and use of their measurement and assessment may inform better interventions and outcomes. Studies across decades have shown that adaptation to enduring cardiac disease and psychosocial recovery from acute cardiac events depends more on psychological than on physical factors^92–94^. The importance of understanding the mind-heart-body connection through constructs such as loneliness and social isolation for clinicians is therefore imperative. Specifically, in the context of this study where the association between loneliness and social isolation and heart failure is recognized, an overview of measurements may inform better interventions and outcomes for people experiencing heart failure.

With the increasing recognition of the association between social isolation and loneliness and heart failure, it is a timely necessity to strengthen the evidence for population and individual health. Population-based decision-making regarding determinants of health relies on standardized and comparable health data to inform practice, guidelines, and recommendations. Individual patient care relies on standardized and quality assessment and measurement of determinants to plan and communicate interventions and evaluate outcomes. This overview of assessment instruments for measuring loneliness and social isolation, promoting standardization and quality assessment.

This study provides an overview of the assessment instruments currently used to measure loneliness and social isolation in people with heart failure. Heart failure is a significant global health concern and therefore warrants such focus. However, given that loneliness and social isolation are widely reported experiences, and are also increasingly identified as determinants of other ill health, this study could inform consideration of the use of measurements in other significant health contexts and chronic diseases such as cancer and diabetes.

One limitation of this review is the exclusion of studies published in languages other than English, so it is possible that some relevant publications were missed. Due to the heterogenous nature of the assessment instruments used to assess loneliness and social isolation across each included study, meta-analysis could not be performed which limits comparison of findings. To ensure a quick and contemporarily relevant review, only three bibliographic databases were searched in the conduct of this review.

To conclude, social isolation and loneliness exert deleterious effects on both mental and physical health, significantly diminishing life satisfaction. The UCLA Loneliness Scale was the most used instrument to assess loneliness and composite measures of network size and frequency of social contacts are most common to assess social isolation in adults living with heart failure. Social isolation and loneliness are established risk factors for elevated morbidity and mortality rates, substantially contributing to a decreased quality of life. Addressing these psychosocial factors is critical not only for improving individual health outcomes but also for reducing the broader societal and economic burdens associated with chronic disease.

## Supplementary information

Supplementary material 1: PRISMA 2020 Checklist

## Data Availability

No datasets were generated or analyzed during the current study.
